# Aggregation-Prone Structural Ensembles of Transthyretin Collected With Regression Analysis for NMR Chemical Shift

**DOI:** 10.3389/fmolb.2021.766830

**Published:** 2021-10-20

**Authors:** Wonjin Yang, Beom Soo Kim, Srinivasan Muniyappan, Young-Ho Lee, Jin Hae Kim, Wookyung Yu

**Affiliations:** ^1^ Department of Brain and Cognitive Sciences, DGIST, Daegu, South Korea; ^2^ Department of New Biology, DGIST, Daegu, South Korea; ^3^ Research Center for Bioconvergence Analysis, Korea Basic Science Institute, Ochang, South Korea; ^4^ Department of Bio-analytical Science, University of Science and Technology, Daejeon, South Korea; ^5^ Graduate School of Analytical Science and Technology, Chungnam National University, Daejeon, South Korea; ^6^ Research Headquarters, Korea Brain Research Institute, Daegu, South Korea; ^7^ Core Protein Resources Center, DGIST, Daegu, South Korea

**Keywords:** transthyretin, nuclear magnetic resonance chemical shift, molecular dynamics computer simulation, protein aggregation, ensemble structure, linear regression

## Abstract

Monomer dissociation and subsequent misfolding of the transthyretin (TTR) is one of the most critical causative factors of TTR amyloidosis. TTR amyloidosis causes several human diseases, such as senile systemic amyloidosis and familial amyloid cardiomyopathy/polyneuropathy; therefore, it is important to understand the molecular details of the structural deformation and aggregation mechanisms of TTR. However, such molecular characteristics are still elusive because of the complicated structural heterogeneity of TTR and its highly sensitive nature to various environmental factors. Several nuclear magnetic resonance (NMR) spectroscopy and molecular dynamics (MD) studies of TTR variants have recently reported evidence of transient aggregation-prone structural states of TTR. According to these studies, the stability of the DAGH β-sheet, one of the two main β-sheets in TTR, is a crucial determinant of the TTR amyloidosis mechanism. In addition, its conformational perturbation and possible involvement of nearby structural motifs facilitates TTR aggregation. This study proposes aggregation-prone structural ensembles of TTR obtained by MD simulation with enhanced sampling and a multiple linear regression approach. This method provides plausible structural models that are composed of ensemble structures consistent with NMR chemical shift data. This study validated the ensemble models with experimental data obtained from circular dichroism (CD) spectroscopy and NMR order parameter analysis. In addition, our results suggest that the structural deformation of the DAGH β-sheet and the AB loop regions may correlate with the manifestation of the aggregation-prone conformational states of TTR. In summary, our method employing MD techniques to extend the structural ensembles from NMR experimental data analysis may provide new opportunities to investigate various transient yet important structural states of amyloidogenic proteins.

## Introduction

TTR is a transporter of the thyroid hormone, thyroxine (T_4_), and holo-retinol binding protein (Ingbar, 1958). It is one of the abundant proteins in human plasma (3–5 μM) and cerebrospinal fluid (0.25–0.5 μM) (Stabilini et al., 1968; Schreiber et al., 1990). In its native state, TTR has a β-sandwich structure consisting of two β-sheets, CBEF and DAGH. In addition, this protein maintains a homotetrameric complex, on which two hydrophobic binding pockets for T_4_ are constructed (Blake et al., 1978). In addition, TTR is also well known for its amyloidogenic propensity, causing several detrimental human diseases, such as senile systemic amyloidosis and familial amyloid polyneuropathy/cardiomyopathy (Westermark et al., 1990; Coelho, 1996). Several biophysical analyses have shown that disruption of the tetrameric complex and subsequent release of monomeric species facilitates aggregation including amyloid fibril formation in TTR (Johnson et al., 2012). Dissociation of amyloidogenic monomers can be caused by several factors, including genetic mutations (Adams et al., 2019), post-translational modification (Poltash et al., 2019; Leri et al., 2020), and proteolysis by proteases, (Mangione et al., 2018; Peterle et al., 2020).

Despite its physiological and pathological importance, the molecular details of TTR aggregation remain elusive. A recent solution-state NMR study revealed that monomerization of TTR causes destabilization of the C-terminal β-stand H, making its neighboring β-stand G more accessible and vulnerable to amyloidogenesis (Oroz et al., 2017). It was previously shown that the TTR (105–115) peptide originating from the β-stand G is highly amyloidogenic (Gustavsson et al., 1991). A recent MD study reported a consistent result in which destabilization of the edge at the DAGH β-sheet, namely the β-stands D and H, is responsible for the amyloidogenic propensity of TTR (Zhou et al., 2019; Childers and Daggett, 2020). Furthermore, a series of computational studies have suggested that the DAGH β-sheet may experience structural deformation to reconstruct aggregation-prone α-sheet-like structures (Steward et al., 2008; Childers and Daggett, 2019). Lim et al. employed solid-state NMR techniques to show that destabilization of the DAGH β-sheet may be caused by the conformational change in the AB loop region (Lim et al., 2016b). From TTR aggregates, they found that the native contact between Leu17 and Pro24 residues in the AB loop was lost, suggesting that non-native distortion of the AB loop may concur with amyloid fibril formation. The structural plasticity of the AB loop has been noted in prior solution-state NMR studies along with unstable structural features of the DAGH β-sheet (Lim et al., 2013; Das et al., 2014). However, there is still a significant gap between direct evidence and theoretical predictions to fully elucidate the molecular details of structural deformation and the resultant aggregation of TTR. In particular, a recent cryo-electron microscopic study of patient-derived TTR amyloid fibrils indicated that TTR should undergo global structural deformation during amyloidogenesis (Schmidt et al., 2019).

Recently, Google DeepMind developed an innovative method, AlphaFold2, which is a machine learning technique to predict the structure of monomorphic and globular proteins from a given sequence (Jumper et al., 2021). AlphaFold2 shows a significant accuracy for globular proteins; however, it is still unknown whether AlphaFold2 can determine the structures of highly flexible proteins, such as intrinsically disordered proteins (IDPs) and metamorphic proteins, or investigate their dynamical features. On the other hand, NMR spectroscopy is a useful tool for investigating structural features of dynamic proteins (Kosol et al., 2013). NMR techniques, including nuclear Overhauser effect (NOE)-based techniques, residual dipolar coupling (RDC), paramagnetic relaxation enhancement, and NMR order parameter analysis, provide long-range or short-range contact information and the degree of structural heterogeneity. In addition, several methodologies using the information of inter-atomic distances or NMR J-coupling have been developed to define the structural ensemble of proteins under physiologically relevant conditions (Meng et al., 2018; Shimomura et al., 2019; Shrestha et al., 2019; Ferrie and Petersson, 2020; Lincoff et al., 2020). Our previous study based on NMR chemical shift, MD simulation, and machine learning technique with multiple linear regression provided a reliable ensemble structure of amyloid beta (Yang et al., 2021), the representative pathogenic IDP (Lin et al., 2019). This method provides the expected conformational states of highly mobile proteins at atomic resolution, which is a novel and rigorous approach to investigate various dynamic features of IDPs and intrinsically disordered regions (IDRs) of diverse proteins.

**FIGURE 1:**
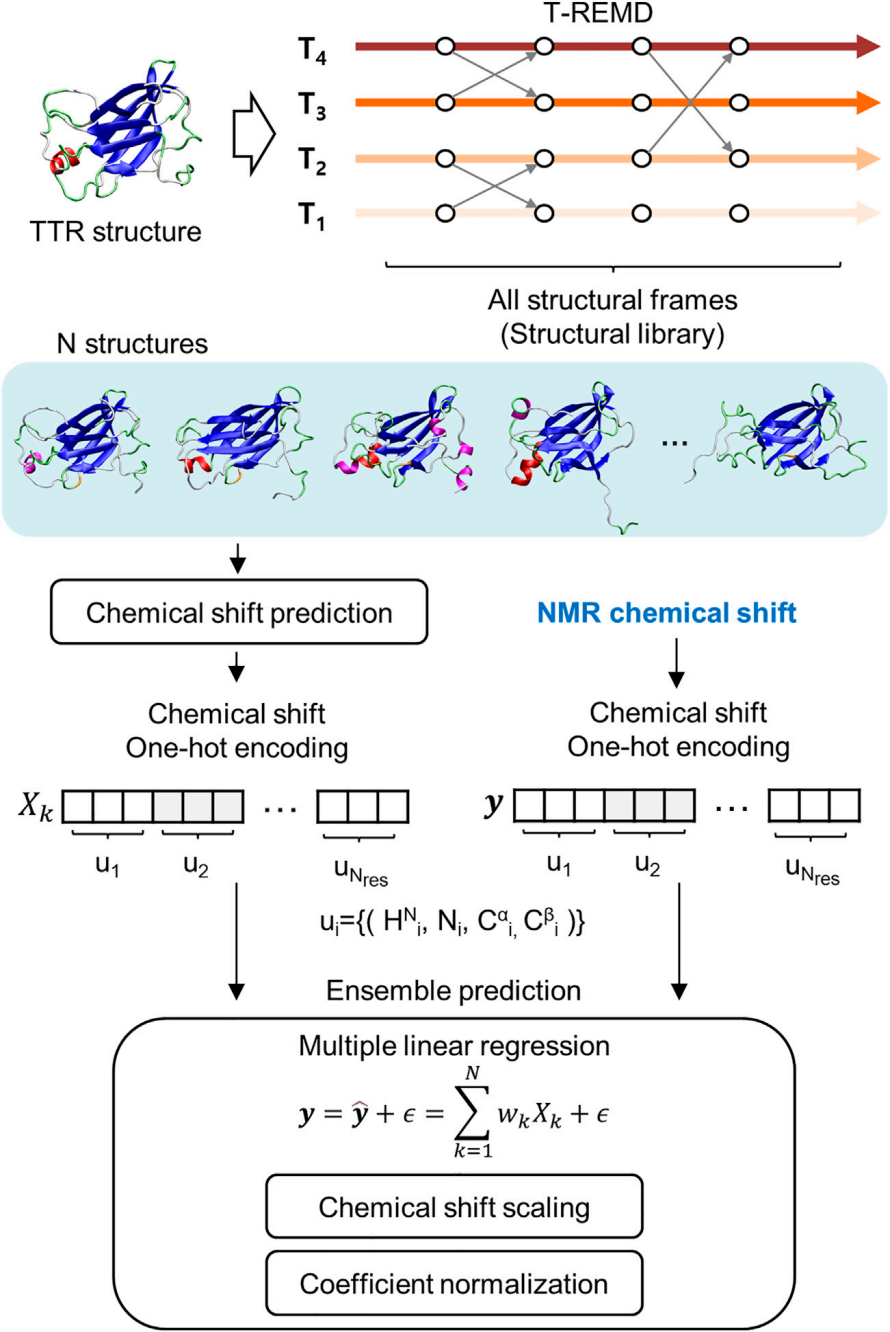
Schematic flow of the regression approach for NMR chemical shift. It includes the generation of structural library, chemical shift prediction, and the main regression scheme for ensemble prediction.

The regression approach used *de novo* structures calculated from MD simulation and the chemical shift prediction algorithm (Figure 1). However, the previous approach mainly using ^1^H_N_ and ^15^N_H_ chemical shift information was insufficient to distinguish the secondary structural features. This is because the distributions of the chemical shifts of the ^1^H_N_ and ^15^N_H_ atoms for the different secondary structures are statistically overlapped (Yu et al., 2011). This study improved the previous regression approach by introducing chemical shift information of the ^13^C_α_ and ^13^C_β_ atoms; the chemical shift of ^13^C_α_ and ^13^C_β_ show significant correlation with the secondary structure of proteins. We successfully introduced the general scaling process into the regression, irrespective of the type of used atom, which increases the accuracy of the regression approach. We revealed the reliable ensemble structure of two monomeric and highly-dynamic variants of TTR, M-TTR (F87M/L110M), and T119M M-TTR (F87M/L110M/T119M), and identified the minor yet reliable ensemble structure using the regression approach. The newly determined M-TTR and T119M M-TTR ensembles provide novel and unprecedented insights into TTR aggregation mechanisms.

## Method

### Experimental Data Acquisition

#### Circular Dichroism Measurement

Human recombinant TTR samples were prepared as previously described in the prior studies (Kim et al., 2016; Oroz et al., 2017). For CD measurement, the concentration of the protein samples was adjusted to 20 μM in a buffer consisting of 50 mM 2-(*N*-morpholino)ethanesulfonic acid (MES) pH 6.5, 100 mM NaCl, and 1 mM dithiothreitol. Cuvettes of 0.5 mm pathlength were used, and the measurement was performed at 25°C. The CD data for protein samples were obtained by subtracting the spectrum of the buffer-only sample.

#### Order Parameter Calculation

The NMR chemical shift data for M-TTR and T119M M-TTR were obtained from BMRB entry ID 25986 and 25,987, respectively (Kim et al., 2016; Oroz et al., 2017). In this study, the deposited chemical shift datasets were first verified using freshly prepared protein samples. Subsequently, the order parameters were calculated using TALOS-N by feeding the experimental chemical shifts of ^1^H_N_, ^15^N_H_, ^13^CO, ^13^C_α_, and ^13^C_β_ (Shen and Bax, 2013).

### Molecular Dynamics Simulation

#### System Preparation

All systems were built using the LeaP program, and all simulations were performed using the AMBER20 MD simulation package (Case et al., 2020). The Amber ff99SBildn force field (Lindorff-Larsen et al., 2010) was used for all simulations. Hydrogen atoms were constrained using the SHAKE algorithm (Ryckaert et al., 1977; Miyamoto and Kollman, 1992). The NMR solution structures of M-TTR and T119M M-TTR were used for MD simulations (PDB code: 2NBO (Oroz et al., 2017) and 2NBP (Kim et al., 2016), respectively). The generation of M-TTR and T119M M-TTR structures with AB loop rebuilding were performed using MODELLER 10.0 (Webb and Sali, 2016). The loop refinement process of the AB loop was applied to the positional restraint which makes the distance between the Leu17 and Pro24 residues to be 20 ± 1 Å. To use ionic strength effects, the salt concentration was 150 mM based on Debye-Hückel screening (Onufriev et al., 2002).

#### Minimization and Equilibration

Each system was minimized with 5,000 steepest descent and a maximum of 2,500 conjugate gradient minimization steps. After the minimization steps, the systems were heated for 10 ns with 2 fs time step from 20 K to each target temperature. The temperature was regulated by a Langevin thermostat with 1.0 ps^−1^ collision frequency.

#### Replica Exchange Molecular Dynamics

To generate an ensemble structure, replica exchange molecular dynamics (REMD) (Sugita and Okamoto, 1999) were performed using the PMEMD program in AMBER20 (Case et al., 2020). Each temperature value for the T-REMD simulation was generated using a temperature generator for REMD simulations (Patriksson and Van Der Spoel, 2008). For M-TTR and M-TTR with AB loop rebuilding, each system with a total of 16 replicas was simulated with a temperature range of 300–507 K. For T119M M-TTR and T119M M-TTR with AB loop rebuilding, each system with a total of 16 replicas was simulated with a temperature range of 300–480 K. During all simulations, exchanges were attempted every 2 ps, and each ensemble was simulated for 1 µs The total sampling time for each system was 16 µs The final average exchange ratios were 14.3, 14.2, 18.9, and 18.8%, respectively.

### Molecular Dynamics Trajectory Analysis

All trajectories were processed and analyzed using CPPTRAJ (Roe and Cheatham, 2013) provided by the AMBER20 package (Case et al., 2020). All snapshots of the trajectories were visualized using VMD (Humphrey et al., 1996). For each mutant TTR, the six trajectories in the three lowest temperature replicas with and without AB loop rebuilding and 20 NMR ensemble structures were used in the contact map and secondary structure analysis. The contact maps were calculated by the distance between Cα-Cα atoms with a threshold of 7.5 Å. The secondary structure was analyzed using the STRIDE program (Frishman and Argos, 1995). The proportion P of secondary structures for each residue is calculated as followed equation:

$$P^{i}\left(\xi \right)=\frac{\sum_{n}^{N}\delta_{\zeta_{n}^{i}=\zeta }}{N},$$

$$Z=\left\{\alpha -\mathrm{helix},\;\beta -\mathrm{sheet},\;3^{10}-\mathrm{helix},\;\mathrm{coil},\;\mathrm{turn}\right\}$$

**FIGURE 2:**
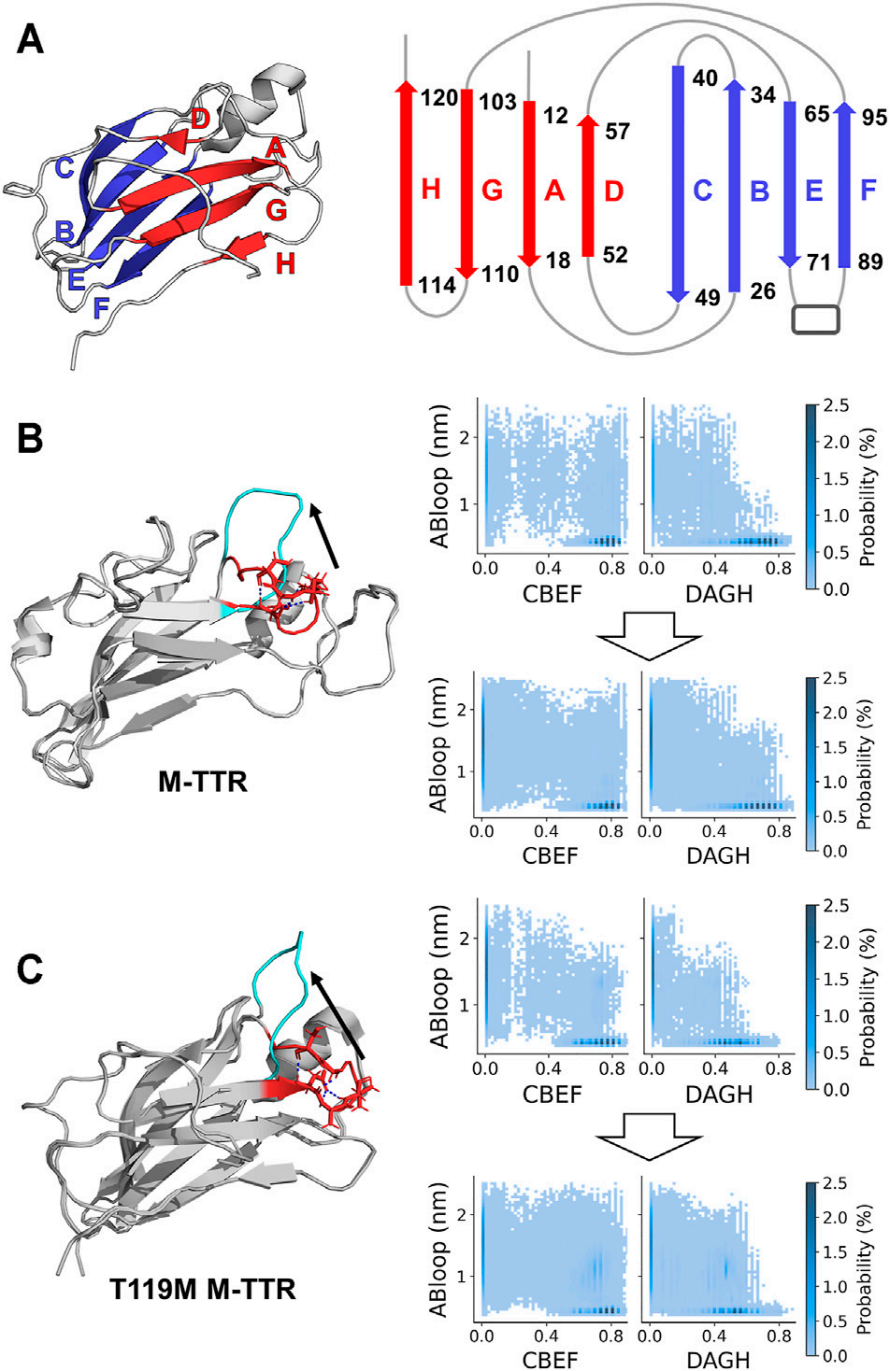
Evaluation of exploring reaction coordinate space and AB loop rebuilding. **(A)** The representation of two main β-sheet structures in the native state of TTR. The CBEF and DAGH β-sheets are colored as blue and red, respectively. These secondary structures are determined by the proportion of β-sheet satisfying more than 50% in the structural library. **(B)** M-TTR (**PDB code:** 2NBO) and **(C)** T119M M-TTR (**PDB code:** 2NBP) **(left)** The comparisons of initial structures between original and AB loop-rebuilt structure. AB loop regions are colored as red and cyan, respectively. Hydrogen bonds within AB loop are colored as blue. **(right)** The density map according to AB loop distance and the ratio of CBEF and DAGH β-sheets. AB loop distance represents the distance between L17 and P24. Density heap maps **(top)** using an original replica with lowest temperature and **(bottom)** using selected structural library which includes the 3 lowest temperature replicas of both original and AB loop rebuilding T-REMD and 20 NMR ensemble structures.

*Z* is a set of secondary structure types. The proportion of specific secondary structure ζ∈Z for residue i is calculated by the total number of conformations which satisfies the secondary structure of *n*th conformation $\zeta_{n}^{i}=\;\zeta \;$ in *N* conformational state of the structural library (*N* = 600,020). And we defined the set Β composed of specific residues which satisfy more than 50% proportion of β-sheet structure for each residue (Figure 2A). Finally, the proportion of β-sheet structure for each conformation is defined as:

$$P_{n}\left(\beta -\mathrm{sheet}\right)=\frac{\sum_{i\in B}^{L}\delta_{\zeta_{n}^{i}=\beta -\mathrm{sheet}}}{\left|\text{Β}\right|}$$

### Chemical Shift Prediction

All visualized results of chemical shift prediction were performed using UCBSHIFT (Li et al., 2020). The other chemical shift prediction algorithms such as SHIFTX2 (Han et al., 2011) and SPARTA+ (Shen and Bax, 2010) were also performed. The chemical shifts of backbone atoms (^1^H_N_, ^13^C_α_, ^13^C_β_, and ^15^N_H_) for proteins were selectively used. The prediction was performed at pH 7.5. The residues whose ^1^H_N_, ^13^C_α_, ^13^C_β_, and ^15^N_H_ atoms were not fully assigned, including proline and glycine, were selectively eliminated. The input structures for chemical shift prediction were obtained from six replicas: the top three lowest temperatures of T-REMD for each original and AB loop rebuilding system, and the published NMR solution structure. Each trajectory gives us 100,000 structures; therefore, a total of 600,000 and 20 NMR solution structures were used for chemical shift prediction.

### Regression Approach

The multiple linear regression of the predicted chemical shifts was based on a previous study on amyloid beta molecules (Yang et al., 2021). We only used the fully assigned residues for ^1^H_N_, ^13^C_α_, ^13^C_β_, and ^15^N_H_ atoms. All regressions were performed using NumPy (Harris et al., 2020) and SciPy (Virtanen et al., 2020) modules, and all visualizations were performed using matplotlib (Hunter, 2007) in Python 3.6.

#### Data Scaling

The scale of the chemical shift varies according to nuclear type. The scaling function f is introduced to solve the scale-difference problem. It is extended by including the additional ^13^C_α_ and ^13^C_β_ atoms compared to the previous method (Yang et al., 2021). The scaling function for each atom is expressed as follows:

f: ℝ→ℝ

$$f\left(x_{i}\right)=\log_{a}\left(\frac{x_{i}-y_{\min}}{y_{\max}-y_{\min}}\right)+1+\theta$$

$x_{i}$ is the chemical shift of a specific atom. $y_{\min}$ and $y_{\max}$ are the minimum and maximum values of the reference chemical shift for the same atom, respectively. a and θ is hyperparameter for minimizing a regression error ϵ . The constants a and θ are determined from the regression for the min-max scaled chemical shift. The basis of min-max scaling is the scale of the reference chemical shift for each atom, similar to the above term in the log function. a is constant in [1.1, 10], irrespective of the atom type. The determining constant θ is based on a previous hyperparameter fine-tuning method for ^1^H_N_ chemical shifts (Yang et al., 2021). Thus, θ is negligible for other atoms without ^1^H_N_ atoms. The hyperparameter tuning process was performed using the parallelized limited-memory Broyden–Fletcher–Goldfarb–Shanno (L-BFGS) algorithm (Gerber, 2020). The scaling function has an obvious inverse function because it is a one-to-one function. Thus, the coefficients of the multiple linear regression after scaling can be equally applied to the original regression without scaling.

#### Multiple Linear Regression

For the interpretation of coefficients as probabilities or appearance of protein structures, we used non-negative least squares (NNLS) regression algorithms that solve the Karush-Kuhn-Tucker (KKT) condition for the non-negative least squares problem with an additional normalization method to make the sum of coefficients to be 1.

$$y=\hat{y}+\epsilon =w_{1}X_{1}+w_{2}X_{2}+\cdots +w_{n}X_{n}+\epsilon$$

$$\overset{n}{\underset{i}{\sum }}w_{i}=1$$

y denotes the experimental chemical shift reference. $\hat{y}$ is the predicted chemical shift among the ensemble of proteins. $X_{i}$ is the chemical shift of the protein structure among the ensemble of proteins. The set $\left\{X_{i}\right\}$ is the generated protein structure library from the MD simulation or other possible methods. $\left\{w_{i}\right\}$ are the coefficients. In this condition, it represents the appearance or probability of each state. n is the number of input chemical shifts from the prediction. ϵ is error value to be minimized. The minimization process was performed using the sequential least squares programming (SLSQP) algorithm in the SciPy optimization module (Virtanen et al., 2020).

#### Feature Selection and Coefficient Normalization

The normalization of coefficients is also used in the previous method (Yang et al., 2021).

$$S={\left\{w_{j}\right\}}_{j=1}^{N}\;such\;that\;w_{j}\geq \varepsilon \cdot w_{m},w_{m}=\underset{w\in S}{\max}S$$

*S* is the set of significant coefficients. ε is a small positive value (10^–5^) as the threshold of selection; the previous study took the same value as a reasonable cutoff (Yang et al., 2021). $w_{m}$ is the maximum element of the set S , which is equal to the maximum element of the entire coefficient set. An additional regression with $\overset{n}{\underset{i}{\sum }}w_{i}=1$ constraint was performed on set S using the SLSQP algorithm.

#### Data Scoring

The scoring method for the regression was based on the coefficient of determination, denoted *R* ^*2*^.

$$R^{2}=1-\frac{\mathrm{RSS}}{\mathrm{TSS}}$$

$$\;\;\mathrm{RSS}=\overset{r}{\underset{k=1}{\sum }}{\left(y_{k}-{\hat{y}}_{k}\right)}^{2},\;\;TSS=\overset{r}{\underset{k=1}{\sum }}{\left(y_{k}-\bar{y}\right)}^{2}$$

RSS and TSS are the residual sum of squares and total sum of squares, respectively. $\bar{y}$ is the mean value of reference. ${\hat{y}}_{i}$ denotes the predicted chemical shift. r is the number of residues in the protein. The total score combining the scores of all the atoms is as follows:

$$R_{\mathrm{total}}^{2}=\underset{\mathrm{atom}\in A}{\prod }R_{\mathrm{atom}}^{2},\;\;\text{A}=\left\{1{\text{H}}_{N},15{\text{N}}_{H},13C_{\alpha },13C_{\beta }\right\}$$

#### Structure Clustering

After the regression, principal component analysis (PCA) and *k*-means clustering were performed for the predicted TTR ensemble. The PCA was based on the Cα-Cα contact map with a 7.5 Å cutoff distance.

### Nuclear Magnetic Resonance Order Parameter Analysis

The NMR order parameter S^2^ was computed using the isotropic reorientational eigenmode dynamics (iRED) method (Prompers and Brüschweiler, 2002) in CPPTRAJ (Roe and Cheatham, 2013). The input ensemble preparation from the regression approach used copying the feature conformations and duplicating each conformation in proportion to its regression coefficient. The final composition of the input ensemble included 1,000 structures. All analyses, except proline residues, were used to consider N-H atom vectors for each residue. The order parameters of the NMR ensemble for both M-TTR and T119M M-TTR were calculated using NMR solution structures.

### Calculation of Chemical Shift Prediction Error and Nuclear Magnetic Resonance Order Parameter Difference

The Euclidean distance in the projection space of the scaling function between NMR experimental data and the prediction of the regression is calculated as follows:

$$f\left(\Delta \delta_{\mathrm{atom}}\right)=f\left(\delta_{{\mathrm{reg}}_{\mathrm{atom}}}\right)-f\left(\delta_{{\mathrm{ref}}_{\mathrm{atom}}}\right)$$

$$\|f\left(\Delta \delta \right)\|=\sqrt{\underset{\mathrm{atom}\in A}{\sum }f\left(\Delta \delta_{\mathrm{atom}}\right)}$$

$\delta_{\mathrm{reg}}$ is the predicted value of the chemical shift from the regression. $\delta_{\mathrm{ref}}$ is a published chemical shift from the BMRB database for M-TTR and T119M M-TTR. $\delta_{\mathrm{atom}}$ is the nuclear-wise chemical shift.

The difference in the NMR order parameter $S^{2}$ between the predicted ensemble from the regression and the NMR ensemble is defined as follows:

$$\left|\Delta S^{2}\right|=\left|S_{\mathrm{reg}}^{2}-S_{\mathrm{ref}}^{2}\right|$$

## Results

### Generation of Structural Library

The aim of our regression approach is to select the important conformations that comprise the protein ensemble in the possible conformation pool. In the free-energy landscape mapping of the conformational states of a system, a globular protein commonly has a sharp, stable state. Therefore, the major ensemble of globular proteins shows a similar conformation, including the backbone positions of the α-helix or β-sheet secondary structures. Thus, the major ensemble of globular proteins can sufficiently represent the entire conformation of the protein. However, this statement is not equally true to dynamic soluble proteins, including IDPs or locally spanned IDRs. The ensemble of the dynamic protein includes many minor state conformations. As a result, the conventional approach often fails to sufficiently reflect minor yet important conformational states. In contrast, our regression approach incorporating NMR chemical shift information can provide a reliable protein ensemble with extended consideration for minor states. NMR chemical shifts collectively reflect appearances and mobile features of all ensemble states at atomic resolution. Therefore, our approach is an accurate and efficient strategy to constrain the MD-based ensemble pool without inadequate removal of relevant conformational states.

To consider important conformations in a sufficient size, we performed MD simulations with enhanced sampling and temperature replica exchange molecular dynamics (T-REMD) to fully explore the conformational space of the protein (Figure 1). The number of input conformations for the regression should be sufficiently large to obtain important conformations. In this process, we refer to a set of possible conformational states as a structural library. T-REMD makes the structural library for M-TTR and T119M M-TTR. However, we considered the possibility of insufficient exploration of the MD simulation because of the large size of TTR. Therefore, we analyzed the amount of exploration of the reaction coordinate space with the proportion of CBEF and DAGH β-sheets and related AB loops. In the present study, we put a particular focus on the AB loop, because it was proposed as an important structural element contributing to the stability of the DAGH β-sheet. A prior solid-state NMR study evidenced that TTR lost the native-like AB loop conformation (Lim et al., 2016b); we consider this observation important and appropriate because it is made with actual aggregates of TTR, not in an un-aggregated soluble state. The contact between the AB loop and DAGH β-sheet is calculated by the distance between Leu17 and Pro24 residues, and we call this term the short AB loop distance. From this analysis, we confirmed that a single simulation could not sufficiently explore the states in which the DAGH β-sheet was stable, and the AB loop distance was large (Figure 2). Considering the previous evidence, we generated the artificial structures of M-TTR and T119M M-TTR to satisfy the AB loop distance, and the average proportion of DAGH β-sheets became large (Figure 2). We used the top three lowest temperature replicas from the T-REMD simulation with the AB loop rebuilding structure for each M-TTR (Supplementary Figures S1). In addition, we considered the NMR ensemble structures of M-TTR and T119M M-TTR as the major ensemble states. After combining all structures into the structural library, we prepared the input structures to map the possible conformational space.

### Determination of Transthyretin Ensemble Using the Multiple Linear Regression for Nuclear Magnetic Resonance Chemical Shift

After generating the structural library, we predicted the NMR chemical shift from each structure in the library. A previous study showed that UCBSHIFT (Li et al., 2020), a chemical shift prediction algorithm, provides better regression quality for IDP-like protein, e.g., amyloid-beta (Yang et al., 2021). UCBSHIFT might be a good prediction algorithm for the mobile C-terminal region of TTR to perform the regression. We prepared the prediction dataset of NMR chemical shifts for all conformations in the structural library using UCBSHIFT. The regression used a total of 600,020 chemical shift sets. The previous study used only the chemical shift values of ^1^H_N_ and ^15^N_H_ atoms for the regression. We have added the chemical shift of the additional ^13^C_α_ and ^13^C_β_ atoms. During the regression, we neglected the partially assigned residues for ^1^H_N_, ^13^C_α_, ^13^C_β_, and ^15^N_H_ atoms, such as proline and glycine residues. The regression solves the minimization problem on the KKT condition with an additional constraint satisfying the sum of coefficients to be one, which uses a non-negative least squares (NNLS) algorithm and additional optimization. To satisfy the revised KKT condition, we interpreted the regression coefficients as the appearance of the conformations in the ensemble. During the minimization of regression, we introduced a hyper-parameter θ to optimize the ^1^H_N_ chemical shift, which depends on the reference chemical shift. The hyper-parameters θ of M-TTR and T119M M-TTR were set to 0.0661 and 0.0431 ppm, respectively. The constants a as the logarithm base in the scaling function were selected in [1.1, 10]. As a result, a values were set to 10. Finally, multiple linear regression analysis provided the probability of each conformational state in the structural library.

**FIGURE 3:**
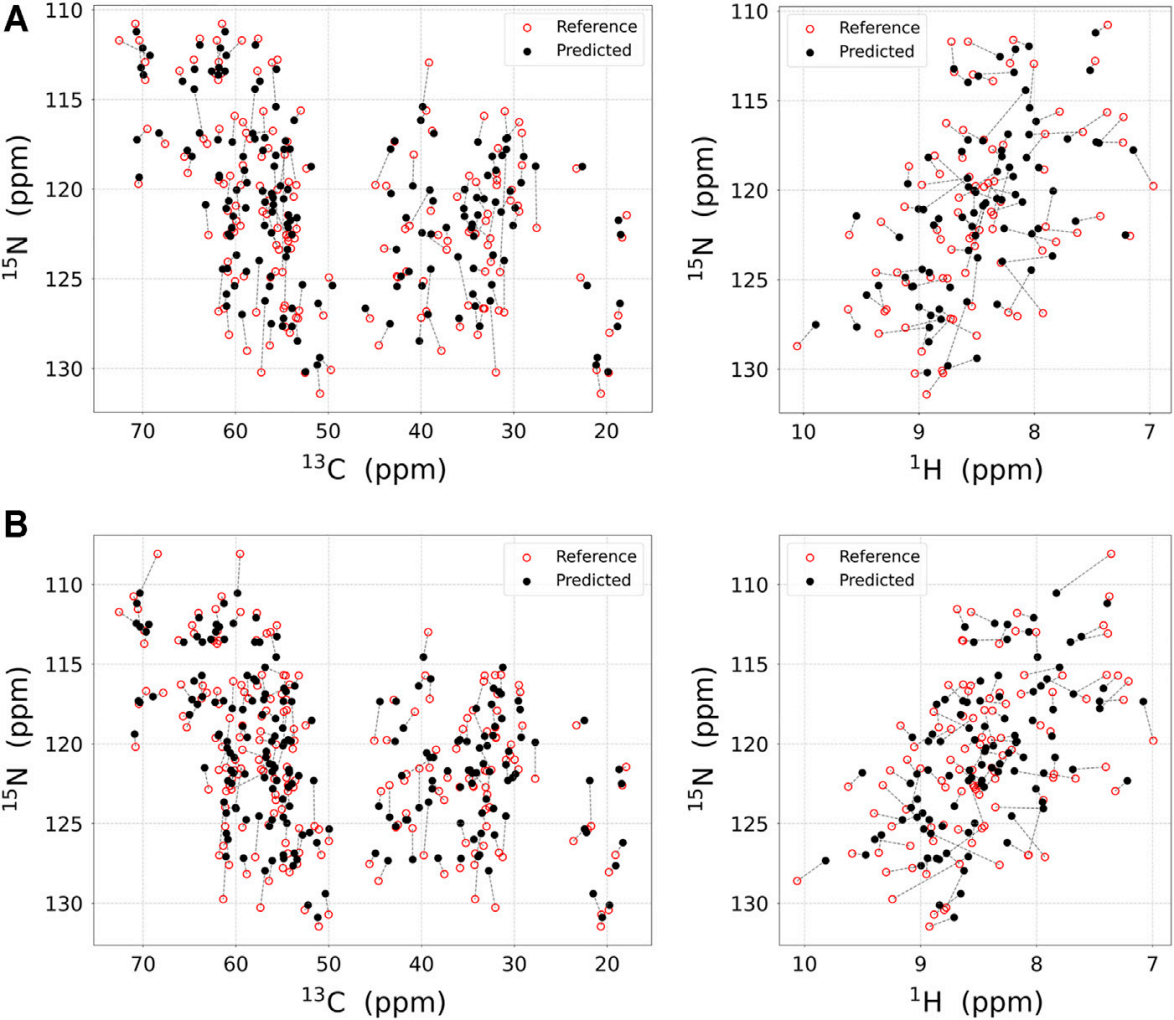
The result of the chemical shift regression approach. Experimental NMR chemical shift **(red open circle)** and predicted chemical shift **(black filled circle)** using regression approach for **(A)** M-TTR and **(B)** T119M M-TTR are displayed in 2D plane. Chemical shifts for the same residues are linked with dotted line. The chemical shift prediction for ^15^N_H_, ^13^C_α_, and ^13^C_β_ atoms (left) and for ^15^N_H_ and ^1^H_N_ atoms **(right)** are respectively plotted. All visualized regression results are obtained with UCBSHIFT. The regression score for each atom is represented in Table 1.

**FIGURE 4:**
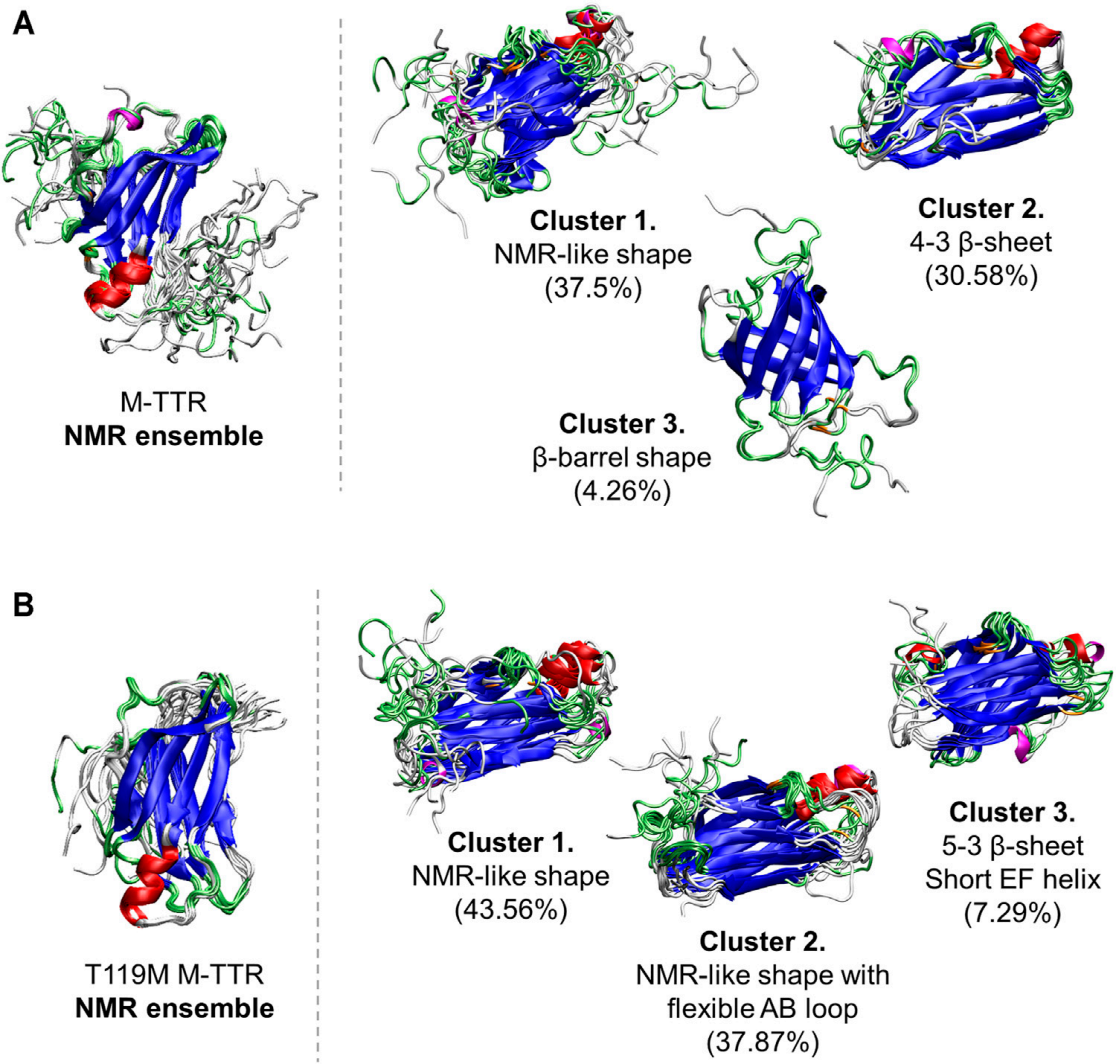
The comparisons of the predicted ensembles with the NMR ensemble. **(A)** **(left)** The NMR ensemble of M-TTR (**PDB code:** 2NBO) has the large amount of fluctuation in the C-terminal region **(right)** There are 3 major clustered ensembles which are predicted from the regression approach. Cluster 1 shows NMR ensemble-like shape with the atomic variability of the C-terminal region. Cluster 2 shows the rigid H β-strand. Cluster 3 shows novel β-barrel-like ensemble which satisfies non-native long distance between L17 and P24 residues. **(B)** **(left)** The NMR ensemble of T119M M-TTR (**PDB code:** 2NBP) has less fluctuation in the C-terminal region **(right)** Predicted ensemble clusters show significant fluctuation in the C-terminal region. Cluster 1 shows the exposure of the G β-strand by released C-terminus. Cluster 2 shows the stationary H β-strand similar to the NMR ensemble. **Color labels;** α-helix **(red)**, 3^10^ helix **(magenta)**, β-sheet **(blue)**, turn **(lime)** and coil **(white)**.

As a result, the regression provided a fitted NMR chemical shift to the experimental reference. We used simple regression analysis to score the regression: the coefficient of determination (*R* ^2^), which is a statistical measure that shows the proportion of variation. The atom scores for ^1^H_N_, ^13^C_α_, ^13^C_β_, and ^15^N_H_ atoms were 0.8737, 0.9686, 0.9963, and 0.9146 in M-TTR, and 0.8864, 0.9370, 0.9975, and 0.9465 in T119M M-TTR, respectively. The ^13^C‒^15^N_H_ and ^1^H_N_‒^15^N_H_ chemical shift plots comparing the experimental and predicted data are shown in Figure 3. The chemical shift error for each atom is also shown in Supplementary Figure S2. After the regression, we compared the original NMR ensemble with the predicted TTR ensemble using regression (Figure 4). The regression provided possible local conformations. We partitioned the conformational ensemble from the regression into clusters using *k*-means clustering in the two-dimensional principal component plane based on the Cα-Cα contact map (Supplementary Figure S3). The major ensembles of both M-TTR and T119M M-TTR include the NMR ensemble conformations as the center of cluster. The second predominant M-TTR cluster show the rigid H β-stand with an increased conformational homogeneity. Notably, the less populated M-TTR cluster exhibit the relaxed β-barrel-like conformation, which satisfies the long AB loop distance between Leu17 and Pro24 residues. These β-barrel conformations have a native-like β-sandwich template including the CBEF and AG β-sheets except that it has a very long D β-strand which connects the CBEF and AG β-sheets into a circular barrel shape. This observation raises an intriguing possibility that disruption of the AB loop may correlate with overall structural perturbation and subsequent aggregation of TTR. On the other hand, the prediction ensembles of T119M M-TTR are similar to those of the previously established with NMR spectroscopy except for a slight difference in the EF loop and the D β-strand. This observation is consistent with the previous studies where T119M M-TTR maintains more homogeneous structural states than M-TTR (Lim et al., 2013; Kim et al., 2016).

Finally, to strictly consider the implication of choosing different algorithms for chemical shift prediction, we performed the regression approach with SHIFTX2 and SPARTA+ (Supplementary Figure S4). Upon comparing the regression quality using *R* ^2^ score (Table 1), we found that the total regression scores for the four atoms are 0.7711, 0.7623 and 0.5116 in M-TTR, and 0.7843, 0.7728 and 0.5959 in T119M M-TTR with the order of UCBSHIFT, SHIFTX2, and SPARTA+, respectively. In the perspective of the regression score, UCBSHIFT is slightly more appropriate than the others for our regression approach, as we concluded in our previous study (Yang et al., 2021).

**TABLE 1:** The regression scores according to the chemical shift prediction algorithms.

	Prediction score (regression)					
	M-TTR			T119M M-TTR		
	UCBSHIFT	SHIFTX2	SPARTA+	UCBSHIFT	SHIFTX2	SPARTA+
^1^ ${\text{H}}_{\text{N}}$	0.8737	0.8429	0.6479	0.8864	0.8793	0.7471
^15^ ${\text{N}}_{H}$	0.9146	0.9363	0.8584	0.9465	0.9289	0.8527
^13^ $C_{\alpha }$	0.9686	0.9693	0.9226	0.9370	0.9491	0.9380
^13^ $C_{\beta }$	0.9963	0.9966	0.9969	0.9975	0.9968	0.9972
Total	0.7711	0.7623	0.5116	0.7843	0.7728	0.5959

### Validation of the Transthyretin Ensemble Predictions

#### Analysis of the Secondary Structures

**FIGURE 5:**
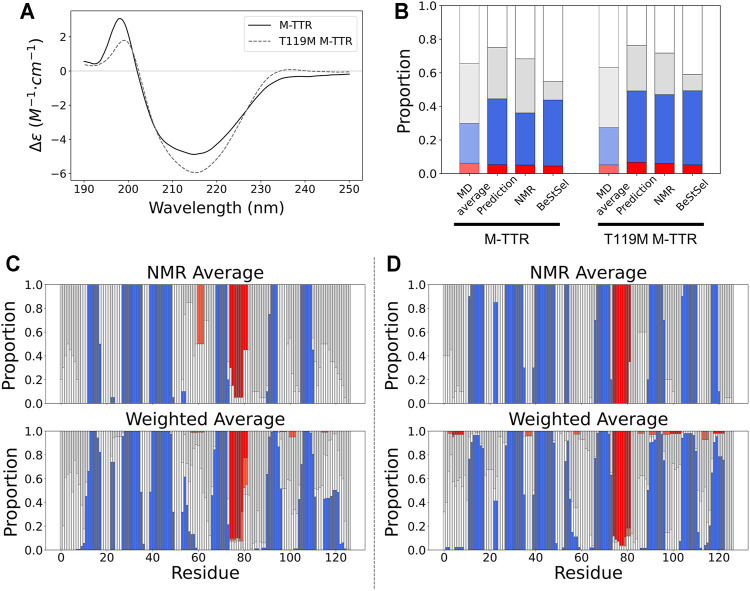
The comparison of secondary structures between NMR ensemble and predicted ensemble from the regression approach. **(A)** The CD spectra for M-TTR **(solid line)** and T119M M-TTR **(dotted line)** **(B)** The secondary structure proportion for all MD trajectories (MD average), predicted ensemble (prediction), NMR ensemble (NMR) and BeStSel prediction using CD spectra (BeStSel). The residual secondary structures of both **(C)** M-TTR and **(D)** T119M M-TTR. **Color labels** α-helix **(red)**, β-sheet **(blue)**, turn **(gray)** and coil **(white)**.

To quantitatively measure the composition of the secondary structures of M-TTR, we used CD spectroscopy with BeStSel (Beta Structure Selection) analysis (Micsonai et al., 2015), which provides the proportion of the secondary structures from the CD spectra using a machine learning approach (Figure 5A). After BeStSel analysis, we compared the secondary structure composition of the predicted ensemble from the regression with the BeStSel results (Figure 5B). Combining all results, we confirmed that our prediction for the TTR ensemble give the most similar results to the BeStSel analysis (Figure 5 and Table 2). In particular, our prediction provided a more reliable β-sheet proportion analysis result for M-TTR than that from the previous NMR ensemble. Subsequent detailed analysis of the results for M-TTR identified that the major difference between our prediction and NMR ensemble is the existence of the C-terminal H β-strand and two short β-strands in residues 21–22 and 54–57. This corroborates that our approach is effective to provide an additional conformational ensemble, which NMR-based ensemble failed to accommodate. In contrast, T119M M-TTR showed that the β-sheet residues from the prediction are mostly consistent with the β-sheet residues of the NMR ensemble. Supplementary Figure provide a comparison between before and after the regression of the secondary structure (Supplementary Figure S5) and the contact map analysis (Supplementary Figure S6). After the regression, the composition of the β-sheet was more manifested than before the regression. This indicates that our regression procedure can efficiently select the relevant and meaningful conformational features.

**TABLE 2:** The secondary structure proportion for each TTR ensemble.

	Secondary structure proportion (%)							
Secondary structure	M-TTR				T119M M-TTR			
	Before regression	After regression	NMR ensemble	BeStSel	Before regression	After regression	NMR ensemble	BeStSel
α-helix	6.2	5.7	5.1	4.5	5.2	6.2	6.0	5.2
β-sheet	23.7	41.5	31.0	39.3	22.2	43.4	41.0	44.1
Turn	35.6	28.3	32.2	10.9	35.7	29.0	24.7	9.7
Coil	34.5	24.5	31.7	45.3	36.9	21.3	28.3	41.0

#### Analysis of the Nuclear Magnetic Resonance Order Parameter

**FIGURE 6:**
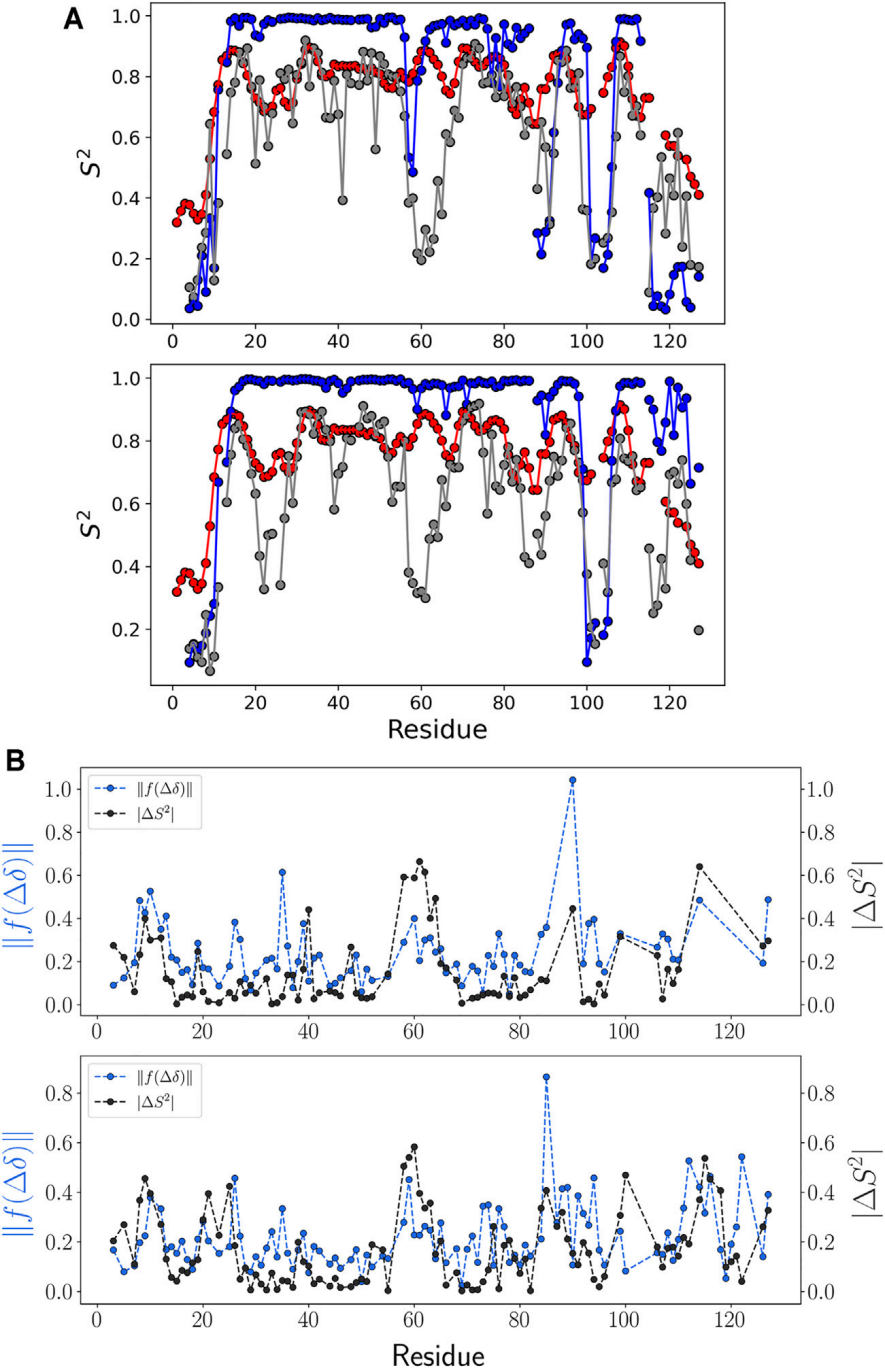
NMR order parameter and chemical shift prediction error. **(A)** NMR order parameter S^2^ for each ensemble of **(top)** M-TTR and **(bottom)** T119M M-TTR is colored as follows: NMR experiment **(red)**, calculation using NMR ensemble **(blue)**, and calculation using the predicted ensemble **(gray)**. **(B)** Comparison between NMR order parameter error and chemical shift prediction error. Errors were calculated by the difference between the regression result and NMR experiment data. Especially, the error calculation of chemical shift prediction was calculated in the projection space by scaling function f .

To verify the predicted M-TTR and T119M M-TTR ensembles, we performed a comparative analysis of the NMR order parameter, representing the amount of fluctuation of the N-H bond vector. We prepared the regression ensemble by copying the feature conformations and duplicating each conformation in proportion to its regression coefficient composed of 1,000 structures. We calculated the NMR order parameter for each residue using N-H vectors, except for proline residues. Combining all NMR order parameter data, we could check the highly conserved regions which compose the specific secondary structures in the solution NMR ensemble (Figure 6A). Our regression ensemble is more consistent to the experimental order parameter except for a few loop regions of both M-TTR and T119M M-TTR. The order parameter analysis for the NMR ensemble showed that the order parameter for the residues around the secondary structure was close to 1. Therefore, the NMR ensemble has more stationary secondary structures and consistent alignment of N-H vectors than the experimental conditions. Each mutant TTR ensemble derived from our prediction was more consistent to its experimental order parameter than the NMR ensemble. In particular, the C-terminal regions of both mutant TTRs maintain the similarities of NMR order parameters between experimental data and prediction. These observations again support the superiority of our approach to reflect the actual structural conformations than the NMR-based ensemble selection.

We verified the correlation between the difference in the NMR order parameters and the chemical shift prediction error (Figure 6B and Supplementary Figure S2). The chemical shift error was calculated using the Euclidean metric in the projection space of the scaling function. The projection space maintains the relative position of each residue in the chemical shift for each atom. From this analysis, we acknowledged that the tendency of the absolute difference of NMR order parameter and the tendency of the chemical shift error in the projection space of the scaling function are similar to each other (Figure 6B), although the quality of regression according to the structural library is robust (Supplementary Figure S7). This observation implies that the chemical shift prediction error is a major limiting factor to have more accurate results with our approach.

## Discussions

Protein aggregation and amyloidosis are among the most critical events associated with various detrimental pathological processes in humans (Chiti and Dobson, 2017). Although several studies have been conducted to understand the related mechanisms, their mechanistic details are still lacking. This is attributed to the highly heterogeneous and dynamic structural states of proteins in their aggregation-prone states (Kelly, 1996; Yang et al., 2021). To overcome these challenges, NMR spectroscopy (Daskalov et al., 2021; Dyson and Wright, 2021) and MD simulation techniques (Prabakaran et al., 2021; Strodel, 2021) are the two major methodologies that significantly contributed to advancing our understanding of the aggregation and amyloidosis mechanisms of various proteins. Indeed, NMR spectroscopy has been a major technique for investigating the mobile structural features of IDPs and amyloidogenic proteins, such as amyloid beta (Crescenzi et al., 2002), tau (Mukrasch et al., 2009), and α-synuclein (Ulmer et al., 2005). In contrast, MD simulations provides physical movements of atoms with the evolution of femtosecond dynamics. It determines the forces and potential energies of interatomic interactions by solving Newton’s equations of motion, which can determine the thermodynamically stable structure of the protein. Several MD-based studies have investigated the dynamics and aggregation mechanisms of amyloidogenic proteins, such as amyloid beta (Tran and Ha-Duong, 2015), tau (Leonard et al., 2021), α-synuclein (Otaki et al., 2018), and other proteins (Meli et al., 2008; Spagnolli et al., 2020).

TTR has been an important target of various structural studies because of its physiological and pathological importance. The native tetrameric structure of TTR is maintained to exert its physiological role as a carrier of thyroid hormones and retinol-binding proteins, whereas the amyloidogenic propensities manifest upon its monomerization (Johnson et al., 2012). Recent NMR spectroscopic studies have shown that M-TTR stabilizes heterogeneous states, in which the C-terminal β-strand becomes highly mobile (Oroz et al., 2017). However, this study could not exclude the possible multiple structural states of this β-stand and the subsequent structural rearrangement. Moreover, it is not clear how its disordered nature correlates with the aggregation-prone property of TTR. It is also noteworthy that the NMR data for determining the M-TTR structural models were obtained under a pressurized condition (0.5 kbar) (Oroz et al., 2017), implying that even more diverse structural heterogeneity may manifest in a physiological condition.

There are many *in silico* methods to determine the structure of IDPs, such as amyloid-beta (Saravanan et al., 2020). Meng et al. (2018) attempted to obtain molecular-level insight and a distinguishable conformational ensemble of the IDP-like protein using MD simulation with single-molecule Förster resonance energy transfer spectroscopy. Other studies used the interatomic distance information from NOEs and RDCs or scalar coupling information to reveal the ensemble of soluble proteins (Meng et al., 2018; Shimomura et al., 2019; Shrestha et al., 2019; Ferrie and Petersson, 2020; Lincoff et al., 2020). Our studies used NMR chemical shift data to determine the conformational state of the protein ensemble. Several methodologies have been developed to use chemical shift data with fragment-based approaches (Cavalli et al., 2007; Nerli et al., 2018; Chandy et al., 2020). Our novel method uses a different approach to extend the experimental observation of NMR spectroscopy using MD simulations. It gives each conformation of the selected ensemble, which is not restricted to any structural constraints. As a result, we can obtain diverse conformational states at atomic resolution in the solution. Moreover, we would like to stress that this regression methodology may be further improved by incorporating additional experimental data, such as secondary structure contents from CD spectroscopy, NOE-based distance information, and J coupling-based torsion angle data. Finally, the present study efficiently expands the exploration range of MD simulation by reflecting the previous experimental observation of the non-native AB loop distance in aggregated TTR. We think that the similar strategy can be effective for MD simulation to explore additional structural abnormality, whose correlation with aggregation propensity was proposed, e.g., the CD loop (Klimtchuk et al., 2018; Dasari et al., 2020), the EF helix/loop (Sun et al., 2018; Ferguson et al., 2021), and the H β-strand (Oroz et al., 2017).

The significant difference between the structural models determined from NMR experimental data and the extended ensembles of M-TTR reported in this study indicates that the C-terminal β-stand, which was determined to be disordered in the NMR models, is still highly mobile. However, it also appears that at least some population of M-TTR may stabilize a native-like β-stand structure in the C-terminal region. NMR structure determination procedures are highly dependent on the accurate analysis of NOE signals, thus limiting the observation of dominant conformations even in the presence of coexisting multiple states. It has been suggested that M-TTR may have several distinctive conformations under native conditions, as observed in the tetrameric conformation of the X-ray crystallographic study (Ulmer et al., 2005), the monomeric conformation of the pressurized NMR study (Oroz et al., 2017), and the distinctive monomeric conformation of the T119M M-TTR NMR study (Kim et al., 2016). In particular, the extended ensembles of this study correlate well with the NMR relaxation dispersion results of WT TTR and M-TTR (Lim et al., 2013; Das et al., 2014), supporting the superiority of our novel methodology for characterizing structural heterogeneity. Finally, the extended ensembles exhibited that non-native loosening of the AB loop accompanies with universal and significant structural perturbation. The AB loop was previously proposed as a region whose structural changes are related to the aggregation of TTR (Lim et al., 2016a). The ensembles indicate that monomerization of TTR may incur structural deformation in the C-terminal β-stand and the AB loop, followed by further structural rearrangement to facilitate amyloid generation.

Our results indicate that T119M M-TTR may have more homogeneous structural states than M-TTR. This is consistent with a series of studies in which T119M substitution increased the overall structural stability of TTR (Hammarström et al., 2003; Lim et al., 2013; Das et al., 2014). In addition, the present ensembles provide a couple of intriguing predictions. First, the C-terminal β-stand harbors reduced yet still significant dynamic features, explaining why T119M M-TTR is more amyloidogenic than WT TTR (Lim et al., 2013). Moreover, our results indicate that the M119 sidechain exhibits several distinctive directions in the ensembles. In previous NMR structural models, the M119 sidechain was positioned inward, suggesting that hydrophobic interaction of the M119 sidechain with other nearby residues may stabilize the C-terminal β-stand structures of TTR (Kim et al., 2016). However, the present structural ensembles show that this residue may have some residual, dynamic features. Subsequent investigation is evidently necessary to appreciate how the mobility of M119 (or T119 of WT TTR) contributes to the aggregation propensity of TTR; yet, our results imply that this residue- or region-specific dynamics may represent structural heterogeneity of TTR in its monomeric and aggregation-prone states. We envision that the models from this study may provide unprecedented insights to design subsequent experimental strategies and to advance our understanding to the aggregation mechanism of TTR.

In summary, these observations support the strength of the current approach in that the calculated ensembles better represent the residual structural flexibility and the amyloidogenic propensity of M-TTR and T119M M-TTR. Although the conformational shape of “real” amyloidogenic species is of great interest to elucidate the mechanisms of amyloidogenesis in detail, its direct experimental observation is challenging due to its heterogeneous and aggregation-prone nature. We expect that our novel methodology may provide a powerful and efficient way to appreciate the dynamic features of amyloidogenic proteins and to reveal the related mechanistic details regarding their physiology or pathology.

## Data Availability

The original contributions presented in the study are included in the article/**Supplementary Material**, further inquiries can be directed to the corresponding authors.
