# Supplementary material for: Aggregation-Prone Structural Ensembles of Transthyretin Collected With Regression Analysis for NMR Chemical Shift
Source: Front Mol Biosci. 2021 Oct 20;8:766830. doi: 10.3389/fmolb.2021.766830 (PMC8568061; doi:10.3389/fmolb.2021.766830)
Supplement: Supplementary file 1 [file DataSheet1.PDF]

Supporting information for

Aggregation-prone structural ensembles of transthyretin collected with regression analysis for NMR chemical shift

Woojin Yang[1], Beom Soo Kim[1], Srinivasan Muniyappan[2], Young-Ho Lee[3,4,5,6], Jin Hae Kim[2,*] and Wookyung Yu[1,7,*]

*Correspondence: jinhaekim@dgist.ac.kr and wkyu@dgist.ac.kr

1. Department of Brain and Cognitive Sciences, DGIST, Daegu 42988, South Korea
2. Department of New Biology, DGIST, Daegu 42988, South Korea
3. Research Center for Bioconvergence Analysis, Korea Basic Science Institute, Ochang, Chung Buk 28119, South Korea
4. Department of Bio-analytical Science, University of Science and Technology, Daejeon 34113, South Korea
5. Graduate School of Analytical Science and Technology, Chungnam National University, Daejeon 34134, South Korea
6. Research headquarters, Korea Brain Research Institute, Daegu 41068, South Korea
7. Core Protein Resources Center, DGIST, Daegu 42988, South Korea

Supplementary figure

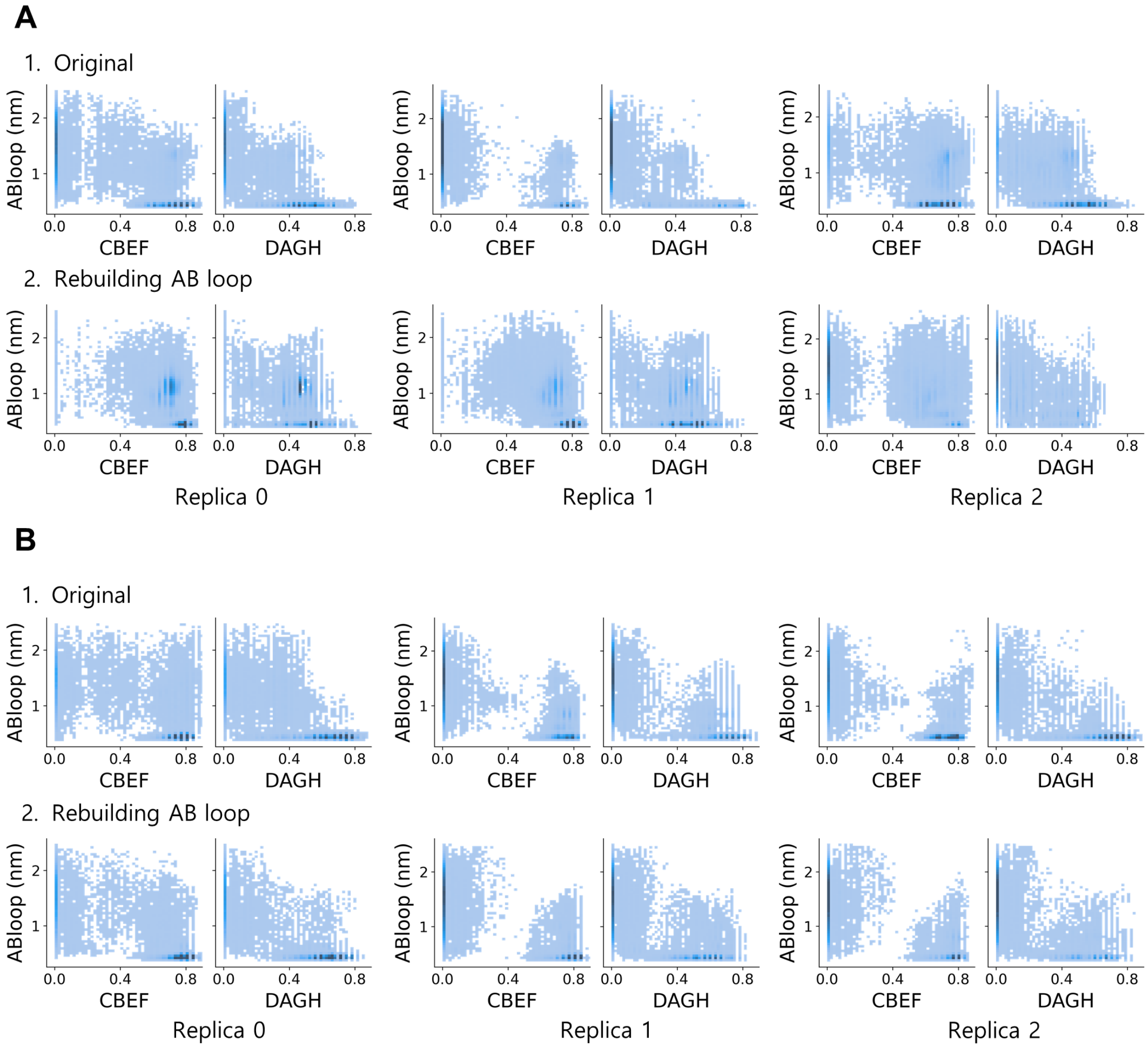

Figure S1. Evaluation of exploring reaction coordinate space. For each replica including original and AB loop-rebuilt MD simulation, the density map of both (A) M-TTR and (B) T119M M-TTR are presented. The color map scale is as same as Figure 2.

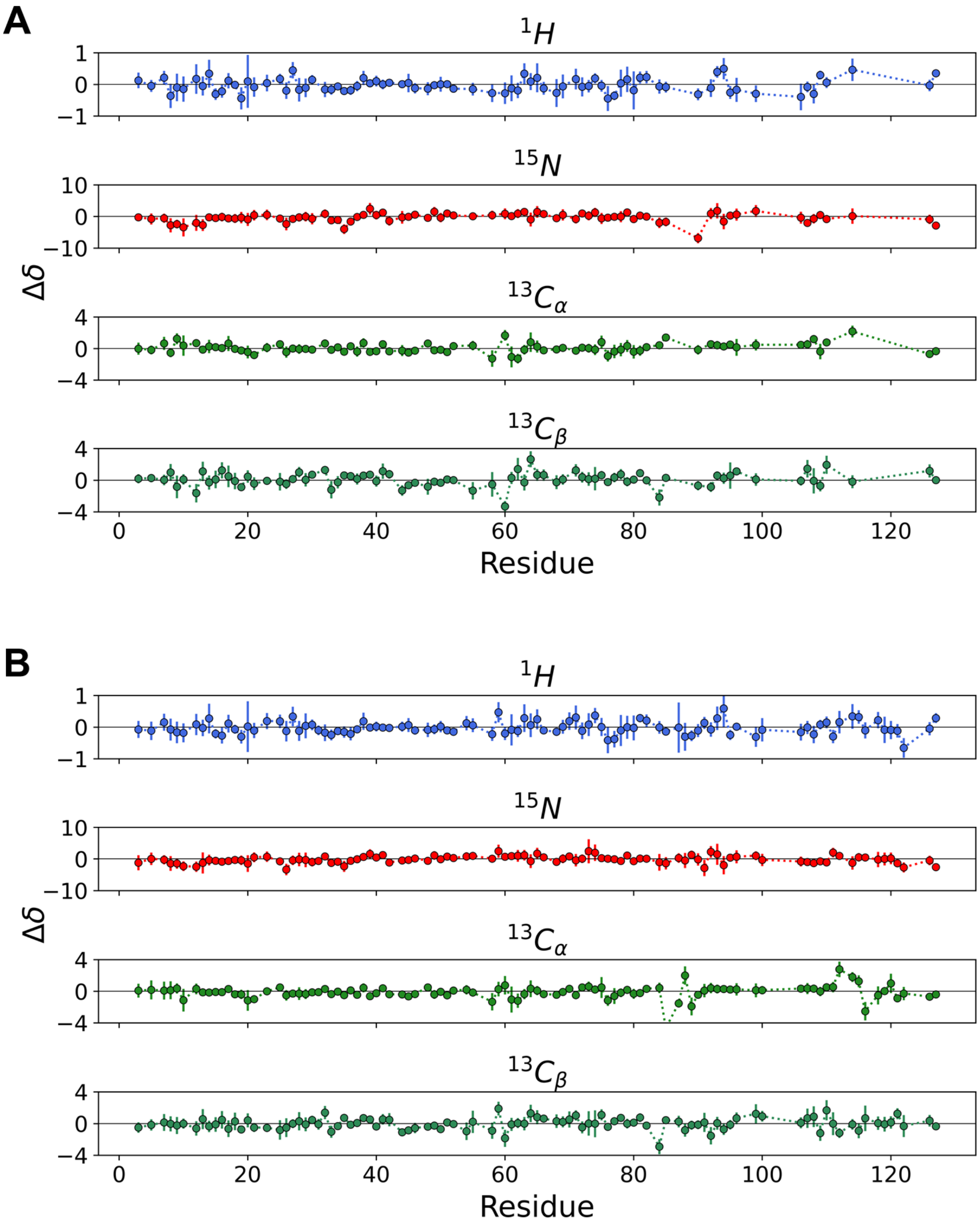

Figure S2. Chemical shift prediction errors. Errors were calculated by the difference between NMR chemical shift data and the predicted chemical shift from the regression. (A) M-TTR and (B) T119M M-TTR. The NMR chemical shift of M-TTR and T119M M-TTR was brought from BMRB (Entry ID: 25986 and 25987, respectively).

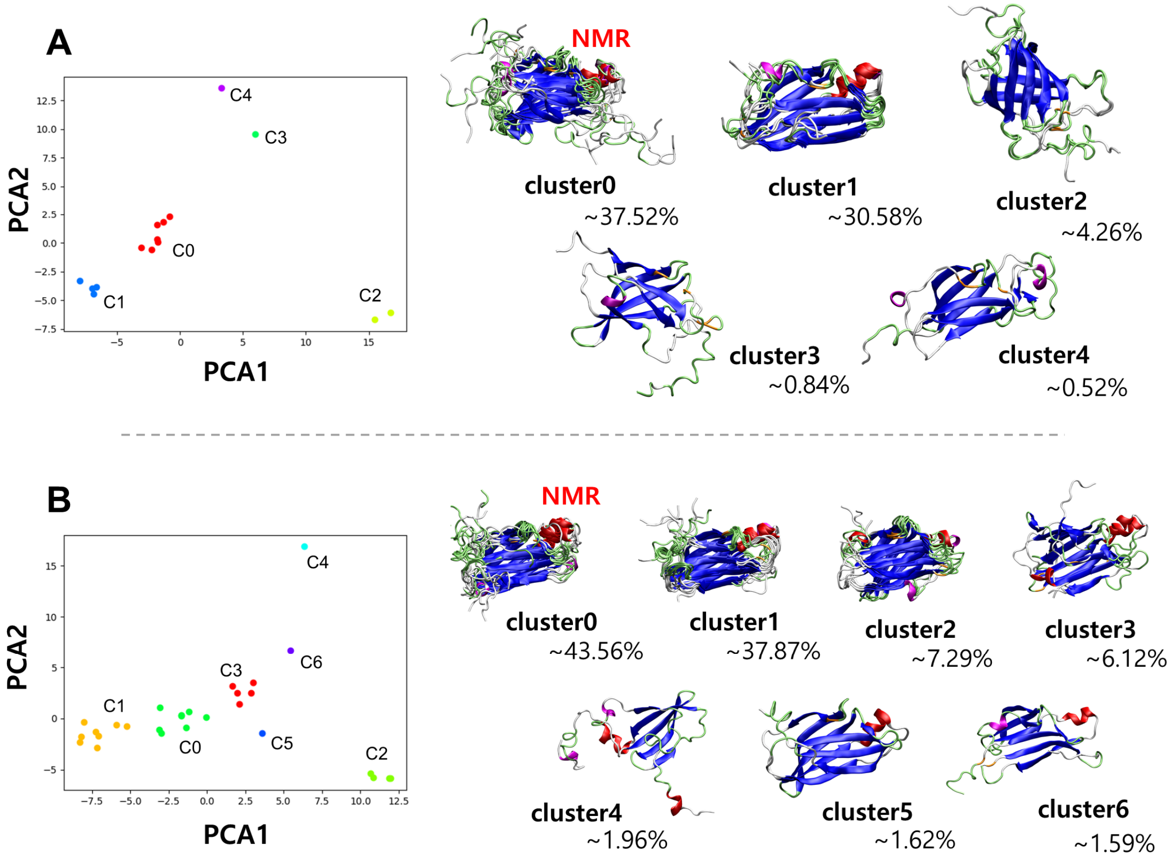

Figure S3. All clustered ensemble from the regression. The ensembles are clustered by *k*-means clustering for (A) M-TTR and (B) T119M M-TTR. (*left*) The results of k-means clustering in PCA plane. (*right*) The secondary structures are colored as: α-helix (*red*), β-sheet (*blue*), turn (*gray*) and coil (*white*). The major clusters with NMR conformations are marked.

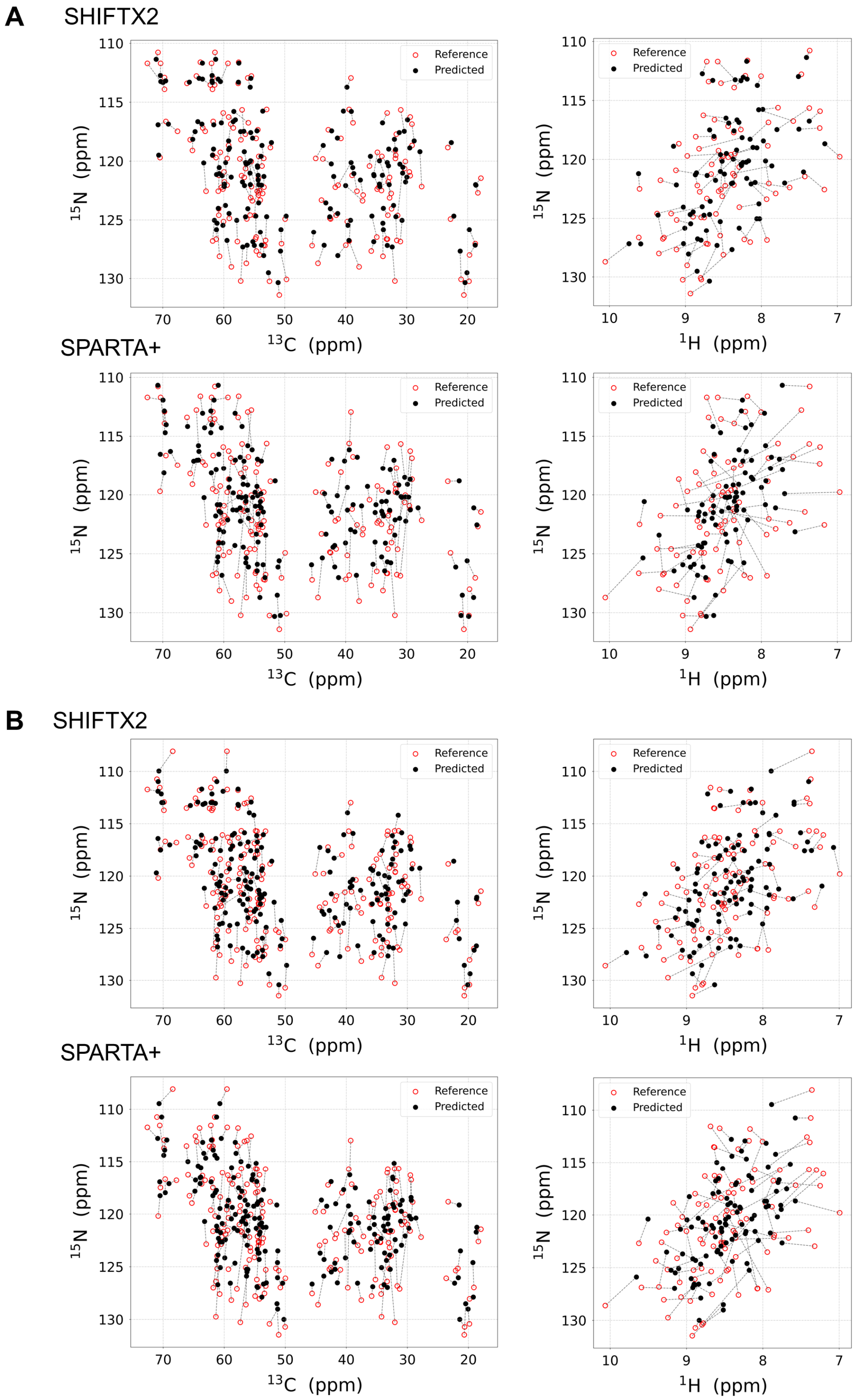

Figure S3. The result of the chemical shift regression with SHIFTX2 and SPARTA+. Experimental NMR chemical shift (*red open circle*) and predicted chemical shift (*black filled circle*) using regression approach for (A) M-TTR and (B) T119M M-TTR are displayed in 2D plane. Chemical shifts for the same amino acid are linked with dotted line. The chemical shift prediction for $^{15}N_H$, $^{13}C_\alpha$, and $^{13}C_\beta$ atoms (*left*) and for $^{15}N_H$ and $^{1}H_N$ atoms (*right*) are respectively plotted. The regression score for each atom is represented in Table 1.

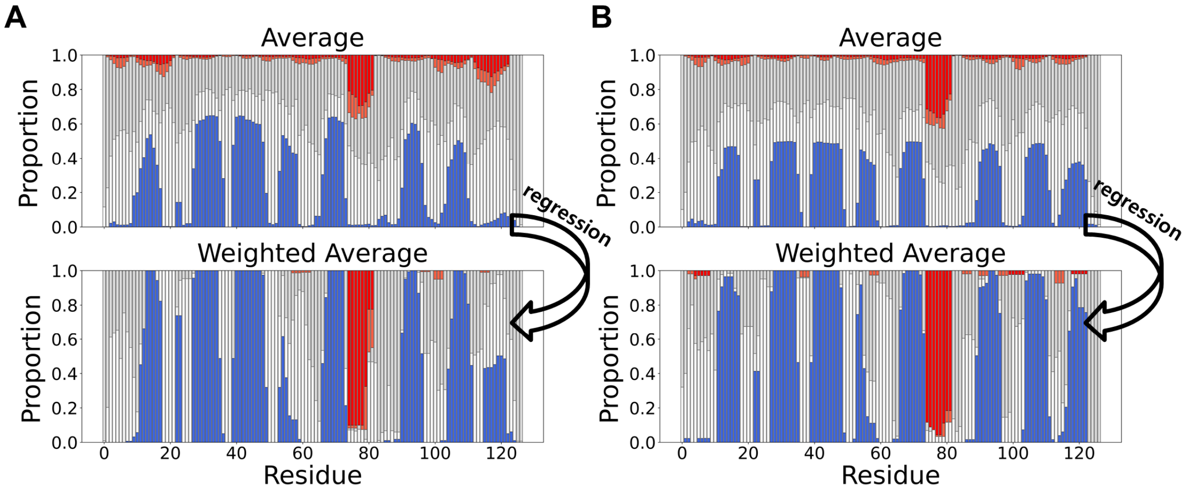

Figure S4. The residual secondary structure proportion before (top) and after (bottom) the regression approach. After the regression, the patterns of β-sheet proportion significantly stand out for both (A) M-TTR and (B) T119M M-TTR. Color labels; α-helix (*red*), β-sheet (*blue*), turn (*gray*) and coil (*white*).

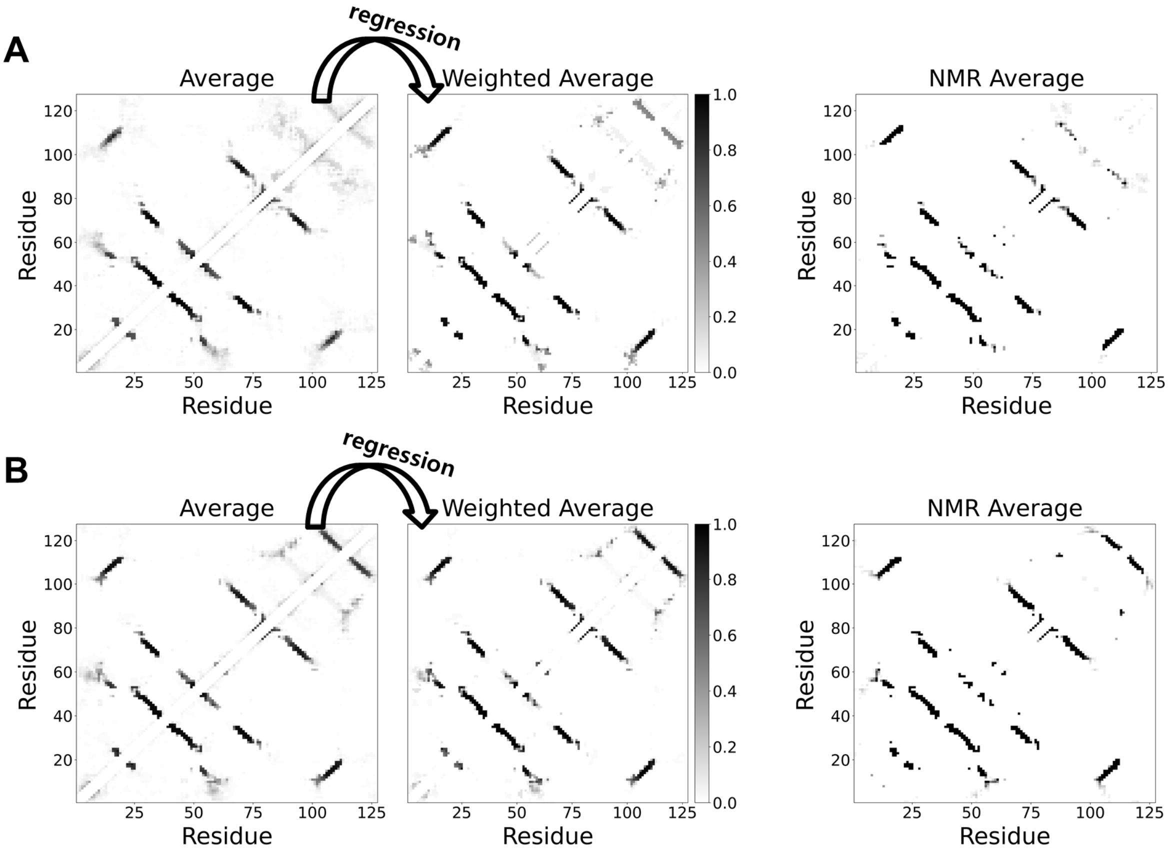

Figure S5. The contacts map before and after the regression approach. (A, B) (*left*) After the regression, the C-terminal contacts patterns significantly stand out for both (A) M-TTR and (B) T119M M-TTR. (*right*) The contacts maps of NMR ensemble are also shown.

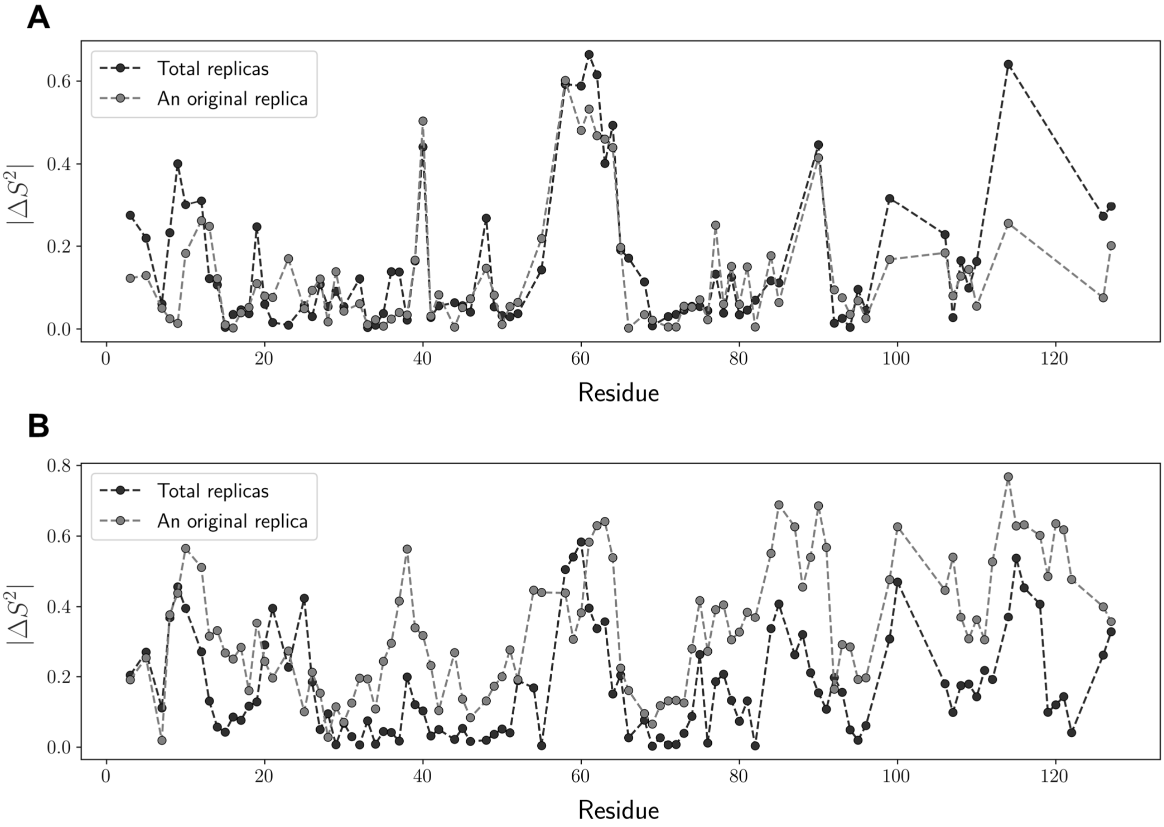

Figure S6. The comparison of NMR order parameter errors. Errors of a replica of original MD simulation and of total replicas including MD simulation with AB loop rebuilding are represented. The order parameter errors are not different between an original replica and total replicas for both (A) M-TTR and (B) T119M M-TTR.
